# Action research study on advance care planning for residents and their families in the long-term care facility

**DOI:** 10.1186/s12904-019-0482-x

**Published:** 2019-11-05

**Authors:** Hsin-Tzu Sophie Lee, Ting-Ru Chen, Chia-Ling Yang, Tai-Yuan Chiu, Wen-Yu Hu

**Affiliations:** 1 Department of Nursing, Tzu Chi University of Science and Technology, No. 880, Section 2, Chien-kuo Road, Hualien City, 97005 Taiwan, Republic of China; 2 Department of Nursing, Chang Gung University of Science and Technology, No. 261, Wenhua 1st Road, Guishan District, Taoyuan City, 33303 Taiwan, Republic of China; 3 Mackay Junior College of Medicine, Nursing and Management, No. 92, Shengjing Road, Beitou District, Taipei City, 11260 Taiwan, Republic of China; 4 Department of Family Medicine, National Taiwan University, No. 1 Chang-de Street, Zhong Zheng District, Taipei, 10048 Taiwan, Republic of China; 5 Department of Nursing, National Taiwan University, No. 1, Sec. 1, Jen-Ai Road, Zhong Zheng District, Taipei City, 10048 Taiwan, Republic of China

**Keywords:** Action research, Advance care planning, Advance directives, Long-term care facility

## Abstract

**Background:**

Research in Taiwan has indicated that advance care planning is rarely undertaken in long-term care facilities. The purpose of this study was to develop an advance care planning interview guideline and care model to facilitate the process of advance care planning for residents and their families in long-term care facilities.

**Methods:**

This study follows an action research design. Cycles of planning, action, observation, and reflection were planned and modified based on the results of interviews with residents and their families as well as meetings with staff. To establish the interview guideline and care model through this action research study, residents and their families were interviewed separately. The researcher subsequently held meetings with staff members to evaluate the results and identify problems during each advance care planning process. This information was synthesised and used to modify the care model for implementation with the next resident–family pair. This process was performed a total of ten times.

**Results:**

This study included residents (*N* = 10), their families (*N* = 20), and medical staff (*N* = 4) at a long-term care facility. The interviews and meetings were audio recorded, transcribed, and subjected to a simple thematic analysis together with the field notes and reflection logs. Four themes emerged from the data related to: opening the conversation with the interview guidelines about the life story of residents; continuing life stories to the quality of remaining years of the residents; gradually changing the topic to the end-of-life care issues; and concluding the conversation by explaining the content of advance directives and hospice care.

**Conclusions:**

The advance care planning care model was implemented following logical thinking from a Chinese perspective. This consisted of opening, developing, changing, and concluding through the views of Confucianism, Buddhism, and Taoism. The research findings indicate that the model successfully facilitated the process of advance care planning for residents and their families.

## Background

Advance care planning (ACP) is a process that includes a set of interventions to promote communication, such as structured interviews, meetings, use of videotapes, and written advanced directives (ADs) [1]. ADs are written instructional health care directives and primarily include three types of documents: a do-not resuscitate (DNR) directive, living will, and durable power of attorney for health care [2]. The purpose of ACP is to clarify and disclose an individual’s care preferences in anticipation of circumstances in which the individual has lost the capacity to make decisions related to end-of-life (EOL) care [3]. Relevant research has indicated that conducting ACP prior to EOL care planning could help seriously ill older adults and their families to better prepare for an upcoming medical crisis, especially among those living in long-term care facilities [4]. However, long-term care institutions in Taiwan and other countries exhibit low ADs completion rates. Foreign studies have indicated that 89–93% of patients expressed a willingness to sign at least one type of ADs [5, 6]. However, nearly 70% of older adult residents in long-term care institutions have not signed any ADs [7]. In addition, Taiwanese studies have revealed that DNR forms are the most commonly provided ADs in long-term institutions, but the signing rate of even these remains lower than 10% [8, 9]. Similar to the results of foreign studies, the current residents of long-term care institutions in Taiwan and their family members demonstrated a high willingness to sign DNR documents (residents 57%, family members 77%) but a low actual signing rate (residents 1.9%, family members 7.1%) [9]. Researchers have also indicated that a wide variety of obstacles impede ACP in long-term care facilities in Taiwan [8–13]. For residents, these include: (1) not knowing what ADs and ACP involve; (2) being unwilling to discuss EOL-related issues; (3) allowing their families to make decisions on their behalf; (4) preferring to allow nature to take its course; and (5) believing that everything is predestined [3–6]. For families, these include: (1) not knowing what ADs and ACP involve; (2) not knowing how to discuss matters related to EOL care with their older family members; (3) fearing that discussion could turn into prophecy and thus being unwilling to discuss death; and (4) fearing being blamed for being unfilial and thus opting for life-sustaining treatments [8, 9]. These findings suggest that the low implementation rate of ACP in Taiwanese facilities is related to the traditional culture of ethnic Chinese people with regards to their reluctance to talk about death and dying [11].

The development of Chinese tradition and culture has been influenced by Taoism, Confucianism, and Buddhism, which have likewise influenced the attitudes and opinions of ethnic Chinese people, particularly on the subject of death. Taoism emphasises people’s connection to natural life forces and proposes that humans should seek harmony with nature rather than attempting to change it [14, 15]. Confucianism provides a basis for the Chinese moral code and behavioural ethics, such as filial piety and familyism. Ethnic Chinese people are traditionally taught that the family should be considered before the individual and that they must properly care for older adults rather than discuss death-related concerns with or in front of them. Regarding Buddhist philosophy, samsara and karma are the two main beliefs. Under the influence of Buddhism, ethnic Chinese people traditionally believe that everything has been predetermined because of karma (cause and effect). The effects of karma can be attributed to an individual’s past life, and the results experienced in the individual’s current life as well as their effects on the next life are referred to as samsara [14–16]. In keeping with these societal mores, findings from the study conducted by Lee et al. [11] demonstrated that only one resident was willing to sign his own DNR directive but that the rest of the participants (*N* = 10, 91%) refused to make independent decisions about EOL care in Taiwan’s nursing home. In this study, residents perceived signing any type of ADs in advance to be unnecessary because they did not intend to go against nature, viewed the family as a decision-making system, and accepted the results of cause and effect.

Apart from the aforementioned influences of religious values on culture, the low implementation rate of ACP in Taiwanese care facilities is also attributable to the cultural influence of logic; that is, the means by which ACP is undertaken contradicts the logical thought process of ethnic Chinese people. The traditional logical thinking model employed by ethnic Chinese people is typified as “opening (起), continuing (承), changing (轉), and concluding (合).” This is the Chinese approach to perceiving the surrounding world and corresponds to the progressive logical process of introduction, elucidation, transition, and summarisation [17]. The manner in which thoughts evolve through this progressive process is referred to as “process philosophizing” [18]. However, the current implementation model for ACP in Taiwan generally begins with the direct delivery of the main topic and ACP-related concepts to patients by health care professionals to receive their opinions. Next, a family meeting is held in which patients and their families acquire an understanding of the concept of ADs through videos or reading, and this is followed by signing ADs [9]. However, when undertaken through the aforementioned process in communities or facilities, the ACP process does not conform to the traditional thought process of ethnic Chinese people. As such, older adults with more health concerns are naturally more resistant to signing ADs [19] and may refuse to participate in ACP [11]. In other words, the current model requires older adults to immediately make treatment decisions after EOL care has been introduced through group health education without first attempting to understand how life experiences may have affected their opinions regarding EOL care.

The aims of this study are as follows: (1) to develop an ACP interview guideline suitable for use in Taiwanese long-term care facilities by older adults through an action research approach and consideration of the traditional views of Confucianism, Buddhism, and Taoism as well as the progressive logical thought process of ethnic Chinese people; (2) to develop an ACP care model to assist residents and their families in the ACP process and improve their understanding of the relationship between signing ADs and having a “good death.”

## Methods

### Study design

This study was conducted using an action research design. Action research combines action and reflection with the participation of others to identify practical solutions to pressing concerns [20]. This methodological approach seeks to bring together action and reflection in participation with others, which is especially relevant in palliative care, given that it requires team work between multiple individuals, particularly in terms of community-based interventions aimed at improving matters [3]. The action group, which comprises voluntary participants, identifies a process for investigating issues and formulates a cycle of planning, action, observation, and reflection. Data generation and analysis are interactive processes that occur continually throughout this type of study. In each cycle, new data are generated. These data are then analysed and used to inform the next phase, with the conclusions implemented in the subsequent cycle. This cycle of action continues until the research group is satisfied that its objectives have been met [20, 21].

**Fig. 1:**
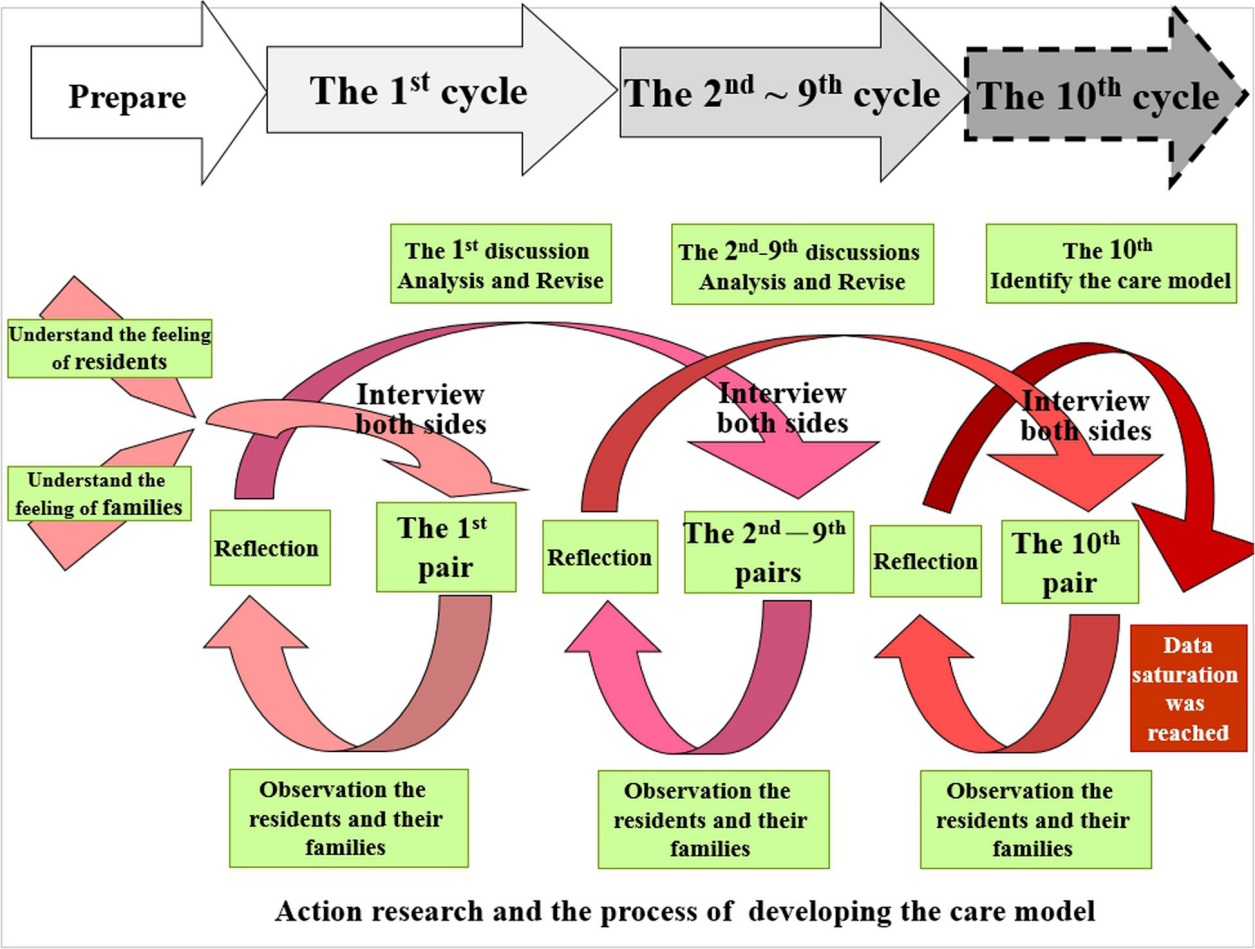
Action research and the process of developing the care model

In the first cycle of action, the first author conducted separate interviews with the first resident and his family and then discussed the results obtained from the interviews, produced in textual form, with the facility’s staff. Based on suggestions from the residents and their families and the outcome of the discussion, the researchers analysed and revised the contents of the interview guidelines and the ACP care model, which were then incorporated into the second cycle of action and applied to the next resident and family pair. This action research process was then repeated until all study participants from the facility found that the interviews helped individuals establish their ACP and agreed on the use of the revised schedule for the establishment. A total of 10 cycles were completed. These qualitative data collection methods were set within an action research approach, which provided a framework for the design and implementation of this study (Fig. 1).

### Setting and participants

The site of investigation was a nursing home in Hualien City. The surrounding region has numerous small-sized nursing homes. The selected nursing home consisted of 42 beds. The residents’ information was as follows: (1) mean age = 80 years old; (2) men-to-women ratio = 1:4; (3) degree of disability (Barthel Index): slight-to-moderate dependency (Barthel score ≥ 60) = 32%; and severe-to-total dependency (Barthel score < 60) = 68%; and (4) Mini Mental State Examination (MMSE) scores ≥24 = 36% (15/42); MMSE < 24 = 64% (27/42). The facility staff comprised 8 nurses, 9 nursing assistants, 1 social worker, and 2 administrative personnel, totaling 20 staff members.

In this study, a purposive sampling method was used and involved three groups of participants, namely the residents, their families, and staff in the nursing home. Potentially eligible nursing home residents, their families, and medical staff were selected using the following inclusion criteria: 1. For residents: (1) residence in the nursing home for > 1 month, (2) age ≥ 65 years and ability to speak Mandarin Chinese or Taiwanese, (3) an MMSE score ≥ 24, and (4) a willingness and ability to share feelings with the researchers about EOL care. 2. For their families: (1) family of a resident that has stayed in the nursing home for > 1 month, (2) must be relatives of the resident and have the ability to speak Mandarin Chinese or Taiwanese, (3) willing and able to share feelings with the researchers about EOL care for residents. 3. The staff participant inclusion criteria were: care providers (including nurses and social workers) who have been in the nursing home for > 1 year and involved with planning or providing daily care to residents. After confirming that the residents, their family members, and staff met the eligibility criteria, the first author then confirmed that they were willing to participate in this study. Participants were added to the sample population until data saturation was achieved.

### Study procedure

Throughout the study period, we adopted purposive sampling and conducted in-depth interviews over a period of 1 year. Before commencing this study, the first author (Lee) worked 2 days per week for 6 months alongside a staff nurse at the long-term care facility to provide care to residents and converse with the residents and their families. After acquiring a general understanding of the concerns that residents and their families had regarding EOL care, the first author conducted a literature review and collaborated with the facility staff to design the interview items for the residents and family members. Subsequently, the first author discussed and confirmed the interview drafts with the fifth author (Hu) before recruiting research participants. Before each interview, the author obtained informed consent from the participants. Additionally, all meetings with the facility staff were audio recorded.

Before initiating the first cycle of action research, the authors decided to separately interview the residents and family members in consideration of the family members’ wishes. Subsequently, the authors held meetings with the facility staff to discuss interview results. After revising the interview guidelines according to opinions from the residents’ family members and staff members, the second cycle of action research was initiated while retaining the separate interview method. After the second cycle of interviews, the first author discussed the results with the facility staff and revised and expanded the interview guidelines. Regarding ACP care model implementation, in addition to retaining the life review and separate interview methods, the first author conducted phone interviews with family members starting from the third cycle of action research. This change was made due to the following reasons: (1) the residents stated that they were willing to express their opinions regarding EOL care to their family members through medical personnel; (2) the family members stated that they wished to share their opinions on the residents’ EOL care with other family members through medical personnel, who would play a mediator role; and (3) the facility staff suggested the first author adopt telephone interviews as the means of interviewing family members to avoid infringing upon the precious visiting time of the interviewees. After completing the interviews, the first author asked whether the residents and family members were willing to share the interview content with other family members. After the completion of the fourth interview, the first author named the interview guidelines for residents and family members as “Life is Like the Seasons” (Table 1) and “Memories of My Dad/Mom/Grandma/Grandpa/Sibling” in a meeting with the staff members, respectively (Table 3). Additionally, after the fourth cycle of action research, the ACP care model was implemented as follows: first, the first author conducted face-to-face interviews with residents to review the course of their life using the analogy “Life is Like the Seasons” (Table 1); next, the first author questioned the residents regarding their opinions on EOL care (Table 2). The first author interviewed the families by telephone. The interview guide entitled “Memories of My Dad/Mom/Grandma/Grandpa/Sibling” was used to interview the families (Table 3). Each interview sessions lasted 30 to 40 min, with 3 to 4 interviews conducted per resident and 2 to 3 interviews conducted per family member. In total, 72 interviews were held, of which 31 were with the residents and 41 were with family members. Furthermore, the first author held multiple interviews because the participating residents from the institution and their family members were unfamiliar with ADs. Some participants even expressed that they had never heard of the term.

**Table 1:** Interview guide, titled “Life is like the seasons,” for opening conversations with residents

1. Childhood is just like the spring	
(1) “When you were a child, what was your dream?”	
2. Teenage years are just like summer	
(1) “Would you please talk about your learning experience or getting a job?”	
(2) “What about your feelings and memories of falling in love with someone?”	
(3) “What about your marriage? Your children?”	
3. Mid-adulthood is just like fall	
(1) “Would you please talk about your family/career?”	
(2) “What makes you proud?”	
4. Senior years are just like the winter	
(1) “What was the most interesting thing in your life after you retired?”	
(2) “What kind of life would you like to have during your old age?”

**Table 2:** Interview guide for the EOL care of residents in the long-term care facility

1. How are you right now?	
(1) “What economic condition are you in?”	
(2) “How do you feel about your health condition now compared with others or last year?”	
(3) “What chronic illness do you currently have?”	
2. What are your options for end-of-life care?	
“What will you worry about when you are at the end of your life?”	
3. “When death is coming…”	
(1) “When death is coming and you can’t eat anything, you will…”	
(2) “When death is coming and you can’t breathe, you will…”	
(3) “When death is coming and your heart stops beating, you will…”	
(4) “When death is coming, will you choose to go to the *Intensive Care Unit* or the hospice unit?”	
4. During the last day of your life, where would you like to stay?	
5. Apart from medicine, what else will you prepare for yourself?

**Table 3:** Interview guide entitled Memories of My Dad [in-law]/Mom [in-law]/Grandma/Grandpa/Sibling

1. “What do you think about the relationship between you and the resident ([grand] father	
[in-law]/[grand] mother [in-law]sister/brother)?”	
2. “Among your family members, who is the closest to the resident? Why?”	
3. “If the condition of the resident becomes serious, what type of care or treatment would you want him or her to have?”	
4. “Do either your family members or the resident not like to discuss topics related to end-of-life care with each other? Why?”	
5. “In what kind of situation would you like to talk about issues related to end-of-life treatment or care together with the resident? Why?”	
6. “If the resident tells your family that he or she wants to sign his or her own ADs, what will your feelings about it be? Will you respect his or her decision?”	
7. “If the resident’s condition becomes serious, do you think that he or she can make decisions about end-of-life treatment by himself or herself? Why?”	
8. “What are your opinions about signing the do not resuscitate documents for the resident?”	
9. “Will the opinions of relatives or neighbours affect your decision about end-of-life treatment or care for the resident?”	
10. “If the resident were dying, what would you feel?”	

### Data analysis

The qualitative inductive content analysis method used was based on guidelines specified by Holsti [22] and Graneheim and Lundman [23]. The transcribed interview content, field notes from visits with each participant, records of each meeting with the long-term care facility staff, and reflection logs were analysed as follows.

**Step one:** The interview transcripts, field notes, meeting records, and reflection logs were compiled and reviewed numerous times by the first author to understand the content.

**Step two:** After statements and response units had been identified, constellations of words, phrases, and sentences relating to the same central meaning were independently allotted into meaningful units by the first, second, and third authors (Lee, Chen, and Yang). This was followed by discussion with and revision by the fourth and fifth authors (Chiu and Hu). These condensed meaning units were compared and organised into subthemes according to their similarities and differences. These subthemes were subsequently presented to all participants, including the facility staff, and revised according to their opinions.

**Step three:** The revised subthemes were extracted from the remaining reviews and allocated into themes. No new themes emerged at this step, and data saturation was achieved prior to analysis of the final interview and meetings with the long-term care facility staff.

**Step four:** An emerging set of meaning units, subthemes, and themes were reviewed separately by each researcher to verify the validity of the findings and conclusions. Themes were also externally validated during this step. The four emergent themes are described in detail below.

#### Ethical approval

The research was approved by the Hualien Tzu Chi Hospital, Buddhist Tzu Chi Medical Foundation, Research Ethics Committee (REC number: IRB103–96-A) prior to commencement and interviews were carried out in accordance with the approved protocol and approved interview paperwork. Statements regarding consent to participate under the Ethics, Consent, and Permissions heading, and another under the ‘consent to publish’ heading were signed, confirming that the author obtained consent from the participants to publish data and report individual patient data.

## Results

A total of 10 participants from a sample of 12 eligible residents, 20 participants from a sample of 24 eligible family members of residents, and 4 participants from a sample of 8 eligible members of the long-term care facility staff participated in this study. The primary reason for excluding 2 of the eligible residents and 4 of the eligible family members was that these 2 residents were going to be relocated to another nursing home following discharge after a period of hospitalization. During the establishment of a care model over the course of this study, eight residents signed the ADs documents on their own, with the assistance of the first author. Their family members (*N* = 16) also all expressed their willingness to follow the residents’ will after being informed by the researchers that their father/mother had signed ADs documents on their own.

### Participant characteristics

Three types of participants were involved in this study, and their respective demographic data were obtained during interviews. The first group of participants comprised residents ranging from 65 to 90 years of age (mean: 79.8 y), most of whom were women (*n* = 80%). Among participants in this group, one had a college degree, six had completed elementary school, and three were illiterate. The average number of chronic conditions among the participants was 3.2, the four most common of which being hypertension (60%), heart disease (50%), diabetes mellitus (50%), and stroke (50%). The religious affiliations of the residents were Buddhist (*n* = 7) and Taoist (*n* = 3). The participants had lived in the nursing home for an average of 5 years. The second group comprised family members of the residents. The participants in this group ranged from 37 to 72 years of age (mean: 54.4 y), and most were women (*n* = 60%). The relationships of the family members to the residents were as follows: child or grandchild (*n* = 16, 80%); child-in-law (*n* = 2, 10%); and sibling (*n* = 2, 10%) (Table 4). The third group comprised the staff of the nursing home. The participants’ age in this group ranged from 28 to 52 years (mean: 39.5 y), and most were women (*n* = 3). The occupations of the staff were as follows: social worker (*n* = 1) and nurse (*n* = 3).

**Table 4:** Participant characteristics of residents and their families

Characteristics	Residents (*n* = 10)	Families (*n* = 20)
	Number (%)	Number (%)
Gender:		
Male	2 (20)	8 (40)
Female	8 (80)	12 (60)
Age:		
35–44 years		2 (10)
45–54 years		4 (20)
55–64 years		10 (50)
65–74 years	3 (30)	4 (20)
75–84 years	3 (30)	
85–89 years	2 (20)	
> 90 years	2 (20)	
Education status:		
College	1 (10)	16 (80)
Elementary school	6 (60)	4 (20)
Illiterate	3 (30)	0 (0)
Religious affiliation:		
Buddhist	6 (60)	12 (60)
Taoist	4 (40)	7 (35)
Catholic	0 (0)	1 (5)
Relationship of primary caregiver to resident:		
(Grand) child (son/daughter/grandchild)		16 (80)
Child-in-law (daughter-in-law)		2 (10)
Siblings (brother/sister)		2 (10)
Chronic diseases:		
Hypertension	6 (60)	
Heart disease	5 (50)	
Diabetes mellitus	5 (50)	
Stroke	5 (50)	
Arthritis	4 (40)	
Kidney disease	2 (20)	
Cancer	2 (20)	
Duration of living in this long-term care facility:		
< 1 year	2 (20)	
1–5 years	6 (60)	
> 5 years	2 (20)	

### Themes

The transcribed content of interviews, the field notes from visits with each participant, reflection logs from the first author, and records of each meeting with long-term care facility staff were analysed as follows.

#### Opening the conversation with the interview guidelines about the life story of residents

**With residents:** The guidelines entitled “Life is Like the Seasons” were used to initiate conversations with the residents, thus providing them with an opportunity to reflect on their life from childhood to late adulthood. When sharing their life experiences with the first author using this approach of life review, all of the residents were willing to describe their relationships with family members and share their life experiences. They often smiled with tears or wept with sadness while discussing joyful or sorrowful memories, and the topic then naturally transitioned to their old age. Some residents even directly disclosed their views on death during this stage, such as Resident 2, who stated immediately after sharing her life experiences from childhood to late adulthood that she did not want to be saved but rather only sought relief.

“I was pretty when I was young and had many suitors. I was in love with a soldier who treated me very well. But my mom said that a soldier’s wife had no future. She said the village head’s family was very rich, and I was forced to marry the son of the village head only to find that he was incredibly ill-tempered. I was beaten often and wanted to divorce him, but I faced resistance from my family. So I endured until he had a stroke and took care of him until he passed away…then I had a stroke myself and they sent me here (the facility)…ah…my life is so miserable and now I just want a quick death. No need to save me (weeps).” (Resident 2).

**With family members:** The interview guidelines entitled “Memories of My Dad/Mom/Grandma/Grandpa/Sibling” were used to begin conversations with family members. In many cases, resident family members indicated that when discussing with the first author either the life moments shared with the resident or the resident’s opinions regarding their family relationships, they felt as though they were beginning to truly understand the resident for the first time. For example, after the review process, Family Member 7 said that she could finally forgive her mother for sending her to a foster family. She indicated that her mother’s life had been incredibly difficult and that she hoped that she could have the experience of a good death. Almost all family members naturally transitioned to the discussion of EOL care through this life review process without expressing fear or resistance to talking.

“I have always felt resentful that my mom gave me away to my aunt’s family because I thought it was because she didn’t like me… now that you have told me that my mom has been feeling sorry about me… because the reason that she had to give me away at that time is that she had to take care of my sick grandparents and my younger brother also fell sick often… knowing this is very important to me, and I cried so much… like I have once again found my mom… indeed, my mom’s life has been very difficult… my dad passed away in an accident long ago, and my mom took responsibility for caring for my grandma and grandpa, who both had strokes. Now it’s her turn to stay in bed and let others do things for her… (sigh)… I just hope at the end when she leaves… she suffers no more.” (Family Member 8).

**In staff meetings:** In each meeting from the 1st to the 10th action research cycle, institution staff members stated that the life review method enabled the residents to narrate and discuss their life experiences, and the topic could be gradually guided to their older years and the current stage. Once the residents expressed their opinions on their quality of life, the researchers could guide them to express their ideas and plans regarding EOL care. Therefore, life review is a suitable method for guiding residents to discuss their opinions on EOL care plans.

“Life review is a suitable option. Both residents and family members feel like they are having a casual conversation and are more willing to discuss taboo topics. After undergoing the life review, the participants were less afraid when asked about their preferred treatment strategy if their disease worsened. The approach of adopting life review methods to start conversations should be retained.” (Staff Member 1–4).

#### Continuing life stories to the quality of remaining years of the residents

**With residents:** When residents mentioned words such as “old” and “useless,” the first author would pose questions regarding the influence of chronic conditions on the residents’ daily lives, the reason for staying in a facility, and their plans for the future. All residents attributed the reason for staying in a facility to their poor health and consequential need to rely on others for care, fear of causing conflicts among their children with respect to caring duties, and fear of becoming a burden and thus ruining the lives of their children. They held exclusively negative attitudes toward staying at the facility or planning for the future and believed that they were just waiting for their last moment to come.

“After retiring, I joined my daughter in the United States and stayed for a few years…but I was still not able to adapt to the life there, so I returned to Taiwan and lived with my son. I have asthma and heart problems. One day, I passed out and woke up unable to walk. My son and daughter-in-law could not care for me due their work obligations. I didn’t want my son to look bad, so I moved to the care facility…people become useless when getting old, are often sick, and feel so uncomfortable…what’s the use of talking about quality of life? Now I’m just counting days, waiting for the time to come, in the hope that God will let me rest in peace (sigh).” (Resident 1).

**With families:** With respect to questions regarding the reason for sending residents to the facility or the quality of life of the residents at the facility, family members all stated that they had to send their family members to the facility. The reasons they stated were the residents’ need for full-time personal care due to health issues and their own responsibilities to work or care for their own family. Although they perceived that the quality of life at the facility was less than satisfactory, they at least had peace of mind and merely hoped that their family members could receive as much relief as possible from the conditions that troubled them.

“I had always lived with my mom since I was little, and the reason she is here is because of what happened three years ago. She fell down on the floor after a stroke, and at that time everyone was at work so there was no one home. Fortunately, the neighbour realised and immediately sent her to the hospital. After that, she came to stay here (at the facility) … although it doesn’t feel as free as home, the facility at least has someone to look after her 24 hours per day… I think that a life without pain is a life with quality when you get old.” (Family Member 12).

**In staff meetings:** Facility staff members opined that upon discussing the residents’ or family members’ opinions on the residents’ quality of life in the institution, both parties would naturally bring up the question of “What if anything serious occurs…” However, interviewing the residents and their family members separately seemed more likely to elicit their actual opinions than interviewing them together.

“We should retain the life review approach to enable residents and family members to discuss life experiences and gradually guide the discussion to their opinions on their current and future quality of life. However, for such discussion to be effective, the residents and their family members must be interviewed separately. Some residents actually wish to return home, but they do not wish to trouble their children. Therefore, they will state that life in the institution is very comfortable and of high quality. This causes family members to perceive that the resident is compromising out of consideration for their family, and this makes them feel remorseful. Once this occurs, it is difficult to shift the discussion to EOL topics or the participants’ actual feelings towards EOL care.” (Staff Member 3).

#### Gradually changing the topic to the end-of-life care issues

**With residents:** When residents began discussing their hopes for experiencing a good death in the future, researchers continued the conversation with a question regarding how the residents would like to be cared if their health conditions worsened one day. At this time, most residents naturally proceeded with the conversation and exhibited no signs of fear or refusal. For example, after completing the process of life review and discussing experiences from childhood to the present, Resident 9, who initially only responded with “never thought about it… don’t ask me… ask my children…” could naturally express her desire to experience a good death when questioned regarding EOL-related matters. She even asked the first author to pass her words on to her children and physicians at the second interview.

“One time, someone came (to the facility) and played a video where they put a large tube into people’s throat… then the electrical shock… and the person got pressed (on the chest) until their bones were fractured… then they asked me if I wanted to be rescued when getting sick… I was only horrified… and could only repeatedly say, don’t ask me and it’s ok to just ask my children… that night I also had nightmares… but last time when you asked me what I planned to do when getting sick, I was thinking… life has always been difficult for me since I was a child. Being a foster child, I later had to marry the beast (her husband) who raped me and who kept chasing other women after we were married. My children and I have also never been on good terms. Now that I’m getting old, after being paralysed by the stroke and having to stay in bed with nappies, they sent me here (cries)… ah… I’m not afraid of death; I just don’t want to die in pain. I don’t want a tube or electrical shock… could you help me and tell my children and the physician?” (Resident 9).

**With families:** When the families were questioned regarding their opinions on the EOL care of the resident, all stated that they actually wanted to know what the resident’s thoughts were but were afraid to ask. They truly hoped that the health care professionals could make queries on their behalves. However, they were all surprised to hear from the first author that the residents wanted to experience a good death but dared to neither talk about it nor sign ADs on their own because of their concerns regarding the responses of their families. After understanding that the residents desired to experience a good death, the family members then expressed their willingness to respect and follow the residents’ wills.

“Mom can only lie in bed with nappies, which is anything but a quality life… if one day she is unwell, should we save her? We should ask her for her opinion. If she wants to be saved, then of course she will be… and if she doesn’t, then we will follow her will… but who dares to ask? She has been convinced that she was sent here (the facility) because we didn’t want her… as her children, how dare we ask what her plan is if she is very sick. (Sigh)… thank you (the first author) for asking on our behalves. Honestly, we felt a deep sense of relief upon knowing that she wanted to experience a good death instead of being rescued… of course her wish shall be fulfilled.” (Family member 17,18).

**In staff meetings:** Facility staff stated that upon asking residents and family members their opinions on the current quality of life in the institution and expectation for life afterwards, almost all residents and family members unanimously and naturally began to share their opinions on death and expressed their wish for a peaceful death. However, both parties feared to shared their opinions with the other party because death is considered a taboo topic; therefore, they both wished that medical personnel could help to convey their opinions to the other party in their place.

“I asked many residents how they felt about living here, and they naturally answered that it felt like they were waiting for death and hoped to pass away in their sleep. Then, I asked them if they wanted to be resuscitated if their conditions worsened. They shook their heads and said that they were already prepared…I also asked their family members for their opinions. The family members also expressed that they wished the resident to pass away peacefully but were afraid that the resident would overinterpret their intentions. Therefore, they were reluctant to initiate disucssion of the topic. The family members stated that it was best to maintain the current situation… Particularly, in cases where the family members hold different opinions on the residents’ EOL care, we medical personnel should notify each family member of the residents’ opinion and guide them in opening a discussion. This would relieve the pressure of making treatment decisions if a resident’s medical condition changed.” (Staff Member 2, 3).

#### Concluding the conversation by explaining the content of advance directives and hospice care

**With residents:** When residents stated their preference for a quick death while sleeping, the researchers took it as an opportunity to introduce ADs from the “good death” perspective. The researcher explained that signing ADs in advance not only provides residents with the opportunity to experience a good death, but that this form of death is also the natural means of passing away advocated by ancient people. Furthermore, the researcher proposed that the circumstances under which an individual one dies has nothing to do with destiny or reincarnation and that the signing of ADs is most crucial. Moreover, the researcher explained that signing ADs in advance also reduces conflict and a sense of guilt among family members. Surprisingly, residents then not only made queries with respect to hospice care but also asked the researcher to explain the process slowly. At the end of the interview, most residents (*N* = 8) actively asked the first author to assist them in the process of signing of ADs.

“Reaching this age… and also living in a place like this… it would be a lie… to say that we never thought about death… if I really can’t make it any further, then don’t save me. I want to tell my children about this, but I fear that it would make them sad… I didn’t know there was this (ADs) to sign… so I thought it was really painful… to get emergency rescue when the final moment came… yet that would be my destiny anyway… I’m just too old… and not smart enough to make the request, so if there’s a person telling me things slowly like this… you ask me and would know that I didn’t get what you mean if I didn’t respond… so you would explain in another way… in this way I could dare to make the request… anyway, it’s ok if you’re the only person knowing that I’m not smart… (laugh).” (Resident 2).

**With families:** Almost all family members would actively make inquiries regarding ADs and services offered through hospice care when learning the opinions of their family members with respect to EOL care. They also hoped that a team would be there to explain the EOL conditions to their family members and help them with the treatment, thereby resulting in less pain.

“After knowing that he/she doesn’t want emergency treatment and wants to pass away naturally… we are now more inclined to dare to think about and plan the next step... but what are ADs? Are they different from do not resuscitate or hospice care? If we don’t want tube insertion, electrical shock, and chest presses, can we still have injections and syringe feeding? ... we hope a medical team can explain this to us. The most important thing to us is helping him/her to pass away smoothly without pain. In this way, later when we look back, we will not have regret from being reluctant to let him/her go.” (Family members 1–5, 7–20).

**In staff meetings:** Facility staff members indicated that the developed ACP interview guidelines were suitable for the institution. The guidelines instructed medical personnel to first guide residents in conducting a life review while introducing EOL caregiving and then to talk about death to introduce ADs documents to the residents. However, if a resident insists that family members sign the documents for him or her, the resident’s opinion must be respected. In such instances, family members become key contact persons for further discussion. Aside from ensuring that a resident’s children understand that the resident wishes to adopt hospice care rather than resuscitation for EOL care, medical personnel must also promote or arrange ADs or hospice and courses related to palliative care for family members.

“We agree that the ACP interview guidelines, which instruct the resident to perform a life review, address the residents’ life plan after aging, and introduce ADs documents after the resident expresses a wish to pass away peacefully, are more suitable for our institution. However, residents who do not wish to sign the ADs documents themselves after the ADs document introduction should not be forced. It is crucial that the family members understand a resident’s actual opinion towards their EOL care. We discovered that upon understanding a resident’s actual opinion, most family members followed accordingly. Our institution should promote ADs-related information to family members or arrange ADs or palliative care courses more frequently. These measures would assist us in discussing relevant affairs with them. Frankly speaking, they may have to use the ADs information one day.” (Staff Member 1, 3, 4).

## Discussion

This study explored how the ACP care model was implemented following logical thinking from a Chinese perspective and was culturally appropriate to facilitate the process of ACP for residents and their families (Table 5). Several salient findings for implementing the process of ACP with residents and their families in the long-term care facility were presented in this study. First, the study findings showed that although many experts had conducted several questionnaire surveys pertaining to EOL care at the facility and given educational courses on EOL and ADs-related documents, surprisingly, when the first author asked the residents whether they were aware of the relevant content of ADs documents, almost all residents (*N* = 9) replied that they had never heard about ACP or ADs-related matters. Furthermore, they even mentioned to the researcher that when the experts had presented documents after delivering the courses and asked if they would like to sign the ADs related documents, most of them would refuse to do so due to uncertainty and fear. This is in accordance with the previous research findings [9, 11, 19, 24]. Currently, ACP-related studies carried out in long-term care facilities in Taiwan are mostly conducted through questionnaires [11, 24]. Among these studies, the findings of Lin showed that residents all expressed extremely high willingness with respect to the idea of signing ADs documents on their own [24]. However, when residents were provided group health education on ADs and hospice care-related concepts and then asked to sign ADs-related documents by themselves on site, it appeared that the older they were and the poorer their health, the more reluctant they were to sign [19]. Taken together, this showed that there was indeed a high willingness accompanied by a low signing ratio with respect to the signing of ADs documents [25–27]. Therefore, the aforementioned research findings presented two important concepts: (1) previous studies conducted using questionnaires to survey older adults in order to determine their willingness to sign ADs-related documents have had limited significance; (2) the conventional approach used to deliver group education regarding ADs and concepts related to hospice care to elderly residents at long-term care facilities has limited effectiveness in promoting knowledge concerning EOL care and ACP participation, and is thus is no longer appropriate.

**Table 5:** The guideline of ACP care model

1. Opening the conversation with the interview guidelines about the life story of residents	1–1 With residents
	• Open the conversation with the interview guidelines known as “Life is Like the Seasons.”
	1–2 With families
	• Open the conversation with the interview guidelines entitled “Memories of my Dad/Mom/Grandma/Grandpa/Sibling.”
2. Continuing life stories to the quality of remaining years of the residents	2–1 With residents
	• When residents mentioned that they moved to the facility because of poor health, questions could be raised regarding the influence of their illness on their current quality of life and expectations for their future life.
	2–2 With families
	• When families disclosed heir reason for sending elderly residents to the facility and their expectations for facility care, explanations could be provided regarding the resident’s current health conditions and expectations for their future life.
3. Gradually changing the topic to the EOL care issues	3–1 With residents
	• When residents mentioned their desire to experience a good death, topics related to EOL care could be discussed.
	3–2 With families
	• Inform families of resident’s decisions regarding EOL care and the reason why they did not discuss this topic with their families.
	• When family members expressed interest in EOL care options, topics related to EOL care could be discussed.
4. Concluding the conversation by explaining the content of ADs and hospice care	4–1 With residents
	• Introduce ADs and hospice care from the “good death” perspective.
	• Explain the relationships among ADs, hospice care, and a good death.
	• Explain the role of ADs, hospice care, and their contents.
	• Explain the significance of signing up from the cultural perspectives of Confucianism, Taoism, and Buddhism:
	- Signing of ADs in advance is the only effective means of fulfilling the goal of naturally experiencing a good death, as advocated by ancient people.
	- The circumstances in which people die have nothing to do with fate or reincarnation. The signing of ADs in advance is most crucial.
	- Signing of ADs in advance is a means of reducing the sense of guilt and conflict in the family.
	• Allow time for elderly residents to ask questions and ensure that they understand the meaning of signing ADs, accepting hospice care, and their perspectives on experiencing a good death.
	• If necessary, assist residents in the signing of ADs.
	4–2 With families
	• Explain and ensure family understanding of ADs and hospice care-related topics such as the ACP process, the content of ADs, and the correlation between hospice care and a good death.
	• Have the medical team set up short-term, mid-term, and long-term care targets with families based on the health conditions of residents.

Second, our study determined that the low ACP implementation rate in facilities in Taiwan was likely related to the approach through which ACP was implemented and the traditional logical thinking mode of the ethnic Chinese. To Chinese people, the traditional approach of directly delivering group education on ADs and concepts pertaining to hospice care to older adults in the community or at facilities [11, 19, 24] contradicted the progressive thinking logic of the ethnic Chinese. Ethnic Chinese people are inclined to employ a “step-by-step” mode of logic thinking with regards to the understanding and acceptance of certain things, especially in the case of unfamiliar or new concepts [17]. In other words, instead of getting straight to the point, ethnic Chinese people typically tend to cognitively approach a topic in a progressive manner [18]. The findings of this study are in accordance with the aforementioned studies: after the first author used life story telling to inspire willingness to talk in both residents and their family members and gradually transitioned the discussion to the EOL topics, all residents (*N* = 10) and relatives [*N* = 20] could naturally, and on their own, express their opinions towards EOL care without reluctance.

Third, this study revealed that the notion of “good death” had a major influence on whether residents and family members were willing to further consider ADs content and be willing to sign ADs. The results indicated that all residents (*N* = 10) and relatives (*N* = 20) wished for a good death, and thus hoped to attain a painless EOL experience and pass away in their sleep. In Chinese culture, having a good death indicates that the resident was a good person in this life and previous lives. This result is consistent with that of previous studies [11, 25, 26]. The British Medical Association defines a good death as “to pass away peacefully and with dignity, to have lived in fulfilment until the last hour” [27]. However, in Chinese culture, a good death holds deeper connotations than simply describing a person dying without pain or with dignity. From the perspective of Buddhism, which has influenced Chinese cultural traditions, if a person does not die peacefully, it indicates that the person’s karma accrued in his/her current and past lives has yet to reach a balance, and the consequences there of may continue to influence their descendants after this person passes away. Therefore, a good death represents a form of blessing to the Chinese people [25, 26]. The study results revealed that when the first author introduced the correlation between signing the ADs documents, hospice care, and good death to residents and their family members from the perspective of good death, the residents actively inquired about the ADs documents and 8 residents even signed ADs with the assistance of the first author. Furthermore, after learning that the residents had completed ADs, their family members all confirmed that they would follow the residents’ decision.

Fourth, the results of this study showed that by replacing the traditional practice of holding a family meeting, healthcare professionals may play a mediating role in concerns related to EOL care by communicating between residents and their families to facilitate the process of ACP. Our findings concur with previous research mainly conducted on Chinese older adults living in the community or in long-term care facilities and their families, which found that older adults and their families lacked knowledge of EOL care, were willing to discuss EOL care but relied on health providers to raise the issue at the right time, and had not discussed EOL care issues within recent years [11, 28, 29]. Taking this study as an example, when assisting residents and their family members in the implementation of ACP procedures, almost all residents proactively stated they preferred to express their EOL care opinions to their family members through medical personnel. In addition, family members expressed a strong expectation that medical personnel should help pass on and coordinate different family members’ opinions on the residents’ EOL care to facilitate reaching a consensus. Otherwise, researchers found that residents and their family members actually already had some ideas about EOL treatment. They only needed help from medical staff to understand exactly what their treatment and care options at the end of their lives were. They also hoped to obtain timely assistance and company from professionals.

## Conclusion

This study revealed that long-term care institution residents, their family members, and facility staff unanimously agreed that guidance from professional medical personnel is necessary when promoting ACP care models in long-term care institutions. Palliative care nurses or nurse practitioners with experience caring for older adults are the most suitable candidates to provide such guidance in current clinical settings. Therefore, the government is advised to plan regional health care networks that categorize institutions into separate regions. A few nurses with the aforementioned qualifications can be hired for each region to be in charge of promoting ACP services in the long-term care institutions within said region. In terms of the personnel expense, we suggest that the long-term care institutions in the government-subsidized network split the cost of hiring the nurses in charge. Once a resident starts receiving palliative care (satisfying the inclusion criteria), the personnel expense can be split between home-based hospice care units and long-term care institutions in the aforementioned network. In summary, the government must support long-term health institutions and palliative care units to promote ACP care models and jointly establish a system of specialized nurse practitioners to guide the caregiving process. Topics of life and death are not merely culturally sensitive; they also affect the emotional exchanges between residents and their families [30]. Therefore, palliative care nurses or nurse practitioners with experience caring for older adults must be present in the institution throughout the caregiving process. Nurses may provide individualized guidance and accompany residents and family members through the last stage of the residents’ life, enabling residents to pass away according to their desired EOL care method. At the end, when residents pass away as they wished to, neither of the two parties will experience regret and both can be at peace.

### Study limitations

This study has several limitations. Because it included participants from a long-term care facility in eastern Taiwan and was the first to implement ACP trials using a long-term care facility as the study setting, it was limited in both its sample representativeness and the manner of interpreting the research findings. First, a limited number of representative cases were collected in this setting. The number of samples and the sampling setting should be expanded in future research to improve the sample quality. Additionally, the specificity and location of the facilities should be considered when selecting long-term care facilities as settings for future studies. Second, the cultural background of the case participants was relatively homogeneous; the study participants were predominantly people of Hokkien or Hakka origin and did not include indigenous people of Taiwan. Therefore, an analysis of the influence of culture on residents’ preferences in terms of EOL care options could not be conducted. Future studies should include residents from different ethnic groups in Taiwan as participants to determine whether any differences exist in this regard.

## Data Availability

The dataset supporting the conclusions of this article is included within the article. However, the raw dataset cannot be shared because of a rule of the Institutional Review Board of the Research Ethics Committee, Buddhist Tzu Chi Medical Foundation.

## Abbreviations

ACP: Advance care planning
ADs: Advance directives
EOL: End of life
